# Coastal Water Quality Modelling Using *E. coli*, Meteorological Parameters and Machine Learning Algorithms

**DOI:** 10.3390/ijerph20136216

**Published:** 2023-06-24

**Authors:** Athanasios Tselemponis, Christos Stefanis, Elpida Giorgi, Aikaterini Kalmpourtzi, Ioannis Olmpasalis, Antonios Tselemponis, Maria Adam, Christos Kontogiorgis, Ioannis M. Dokas, Eugenia Bezirtzoglou, Theodoros C. Constantinidis

**Affiliations:** 1 Laboratory of Hygiene and Environmental Protection, Medical School, Democritus University of Thrace, 68100 Alexandroupoli, Greece; atselemp@med.duth.gr (A.T.); egiorgi@med.duth.gr (E.G.); elle6_64@hotmail.com (A.K.); johnolbas@hotmail.com (I.O.); ant99tselemponis@gmail.com (A.T.); admaria@windowslive.com (M.A.); ckontogi@med.duth.gr (C.K.); empezirt@yahoo.gr (E.B.); tconstan@med.duth.gr (T.C.C.); 2 Department of Civil Engineering, Democritus University of Thrace, 69100 Komotini, Greece; idokas@civil.duth.gr

**Keywords:** coastal water, machine learning, *E. coli*, predictive modelling, pollution

## Abstract

In this study, machine learning models were implemented to predict the classification of coastal waters in the region of Eastern Macedonia and Thrace (EMT) concerning *Escherichia coli* (*E. coli*) concentration and weather variables in the framework of the Directive 2006/7/EC. Six sampling stations of EMT, located on beaches of the regional units of Kavala, Xanthi, Rhodopi, Evros, Thasos and Samothraki, were selected. All 1039 samples were collected from May to September within a 14-year follow-up period (2009–2021). The weather parameters were acquired from nearby meteorological stations. The samples were analysed according to the ISO 9308-1 for the detection and the enumeration of *E. coli*. The vast majority of the samples fall into category 1 (Excellent), which is a mark of the high quality of the coastal waters of EMT. The experimental results disclose, additionally, that two-class classifiers, namely Decision Forest, Decision Jungle and Boosted Decision Tree, achieved high Accuracy scores over 99%. In addition, comparing our performance metrics with those of other researchers, diversity is observed in using algorithms for water quality prediction, with algorithms such as Decision Tree, Artificial Neural Networks and Bayesian Belief Networks demonstrating satisfactory results. Machine learning approaches can provide critical information about the dynamic of *E. coli* contamination and, concurrently, consider the meteorological parameters for coastal waters classification.

## 1. Introduction

Water is vital for sustaining life in various ecosystems and in humans. However, water sanitation and quality criteria must be met in order for water to be consumed. Water pollution is essential in developed and developing countries, while water deterioration, due to pathogenic bacteria, can be lethal. Therefore, quality criteria based on microbiological and chemical analysis have been established. According to the World Health Organisation, unsafe or inadequate drinking water can lead to 829,000 people globally dying due to diarrhoea. In the European Region, seven people die daily due to unsafe water, sanitation and hygiene. Among the pathogenic bacteria that cause water-related disease is *E.coli* [1,2].

Swimming in seawater that has poor microbiological quality can be dangerous for swimmers and visitors in these areas. During the summer season, the ecological conditions of beaches and seas undergo changes as they are affected by microbial contamination caused by human activities. The presence of faecal microorganisms, including *E. coli* and enterococci, is a significant indicator of the microbial contamination, which is primarily caused by human-related factors like pets and small ruminants, for example [3]. 

The detection of these, and the sanitary management of coastal areas, require an im-mediate lack of measures in case specific pathogenic microorganisms are detected. Health authorities should provide reliable measurements to make decisions, even on the same day, about the state of the coastal areas. Contaminated coastal water could cause swimming-related illnesses such as gastronomic disorders, upper respiratory infections, eye infections, etc. Developed countries, such as the USA, have quantified the economic impact of such incidents on their health system; sick leaves in California cost about 3.5 billion dollars [4].

The Directive 2006/7/EC seeks to update the provisions of Directive 76/160/EEC by simplifying the monitoring and management of bathing water quality. The Directive classifies bathing water into four quality levels: (a) Insufficient, (b) Adequate, (c) Good and (d) Excellent. Concerning *E. coli* concentration, European and national legislation requires specific limits. For the quality category “Excellent”, *E. coli* should not exceed 250 cfu/mL, while for the ‘good’ and ‘sufficient’ coastal water categories, the critical value is 500 cfu/mL, considering the percentiles evaluations. In addition, the standard laboratory procedures regarding the microbiological analysis of the samples should always comply with the preferred methods of ISO, namely: ISO 9308-3 or ISO 9308-1 (European Commission Directive 2006/7 (Council Directive 2006/7/EC. https://eur-lex.europa.eu/legal-content/EN/TXT/?uri=CELEX%3A32006L0007&qid=1673547450307 (accessed on 28 October 2022).)).

Recently, with the increase in computational power, the use of new mathematical models has also increased in the field of water ecosystem management. Environmental and health authorities use algorithms and prediction models to anticipate water pollution issues of chemical and microbiological origin. Machine learning algorithms, a subdomain of artificial intelligence that has been exploited in water resources management, include artificial neural networks (ANNs), fuzzy inference system (FIS), evolutionary algorithms, the support vector machine (SVM), the random Forest (RF) and the decision tree (DT), among others. Their implementation and prediction ability have been tested in drinking water quality, seawater desalination, liquid waste management, groundwater, coastal water management and for coastal hydro-environment management [5,6].

Prediction models serve as rapid decision-making tools that are able to incorporate real-time data, enhancing the speed and accuracy of predictions. These models leverage their computational capabilities and encompass multiple meteorological, climatic and hydrodynamic factors, including water temperature, air conditions, turbidity and precipitation. Researchers have put forth statistical models and machine learning algorithms to predict the aforementioned pathogens [7,8,9,10]. 

The integration of predictive models with the advancements in information technology and field-specific technologies, such as the continuous monitoring of sensor-generated data for water volumes, holds significant promise. The use of cyber-physical systems and various sensor-based water quality monitoring systems, e.g., physical monitoring sensors, optical remote sensors and real-time monitoring sensors, in conjunction with algorithms like neural networks, support vector machines, and long short-term memory (LSTM) deep neural networks, can provide reliable solutions for water quality monitoring, management and governance. These combinations can also contribute to effectively managing data related to water quality monitoring, including time series, models, uncertainties and errors, while simultaneously improving the predictive capabilities of machine learning models [11,12,13].

Μicrobiological, chemical and physicochemical parameters are often integrated into corresponding indicators to create a scientific basis for categorising water quality. Drinking water, as well as other ecosystems such as rivers, are studied using these indicators. In addition, predictive models can be applied by assessing the level of water contamination and its corresponding quality level in a formulated water quality index [14].

The capability of predicting coastal water quality through machine learning methods is not restricted to model pathogens but also to eutrophication phenomena. The goal of such approaches is to assess the Chl-a concentration. These incidents affect the anthropogenic, the urban and the marine environment. Support vector machines, random forest and artificial neuron networks have been successfully implemented towards algal predictions [6,15]. 

Similarly, other research focused on the phenomenon of eutrophication, through the creation of a prediction tool, with the help of a linear regression and artificial neural network. The variable to be assessed was the dissolved oxygen of a coastal lagoon in Murcia, Spain. In this study, the artificial neural network model had higher accuracy [16].

The timeframe in which individuals can engage in bathing activities largely relies on local regulations and weather conditions. Typically, in Greece, this period extends from May to September. Moreover, Greece boasts one of the world’s most extensive coastlines, with numerous beaches attracting a high volume of tourists. This emphasises the significance of the tourism industry for both the regional and national economies. However, the contamination of coastal waters with faecal matter has given rise to public health concerns, resulting in a decline in water quality and placing significant strain on environmental and public health agencies. 

## 2. Related Work

Much research confirms the link between pathogenic microbes, mainly coliforms, in aquatic ecosystems and human diseases. Although no single pathogenic microorganism is considered the absolute indicator for categorising the quality of marine ecosystems, nevertheless, enterococci, *E. coli* and bacteriophages are considered good indicators. The potential presence of only the above pathogens confirms the necessity of a modern surveillance program of marine ecosystems for their microbial quality and the presence of pathogens, let alone in those waters used for recreation and swimming [17,18,19]. 

In the 1980s, the correlation between the presence of *E. coli* bacteria and swimming-related illnesses, including gastrointestinal and skin diseases, was established in Hong Kong. As a result, *E. coli* became widely recognised as an indicator for assessing the microbial quality of water. Subsequently, the detection of this particular organism was incorporated into all monitoring programs aimed at evaluating the quality of bathing waters [20].

In addition, faecal-origin contamination in aquatic ecosystems originating from anthropogenic activities also carries the risk of antibiotic dispersion in surface waters. Water quality surveillance, mainly in point and non-point pollution sites, rivers, river streams, deltas and marine ecosystems, could provide more information on the circulation of antibiotic-resistant genes in the environment and the impact of water quality in microbial abundance and marine biofilm formation [21].

The quality of water reflects the quality of the environment which, in turn, affects the human perception of the quality of life. The quality of human life also includes variables related to the quality of water in coastal areas and these are often considered as indicators that reflect the perception that humans have of the environment in which they live—social and cultural—which, in turn, is related to quality of life on a psychological and physical level [22].

Despite the tremendous economic value of Greek coasts and their contribution to the national economic and cultural capital, research on their health status is not sufficiently reflected in the scientific literature. In the 1990s, four studies focused on the quality of surface waters. The microorganisms examined were *Salmonella* sp., *Yersinia* sp. and *Campylobacter* sp. in rivers and lakes. In research referring to coastal waters, *Ε. Coli* is mentioned, but no further documentation is currently present. Finally, in the most comprehensive research of this decade, the presence of enteroviruses and adenoviruses in marine samples was studied concerning coliform bacteria in South Western Greece. Almost 12% and 50% of samples from different regions did not meet EU microbiological limits for coastal water quality [23,24,25,26].

Over the same period, four separate studies investigated the microbiological quality of swimming waters and *E. coli* was detected in six samples from the Athens area. Furthermore, in a monitoring program on the coast of north-western Greece for a 4-year period, from 1996 to 2000, in all samples collected from May to September—that is, the entire monitoring period of coastal waters—*E. coli* was detected. Furthermore, a study on the microbiological analysis of coastal water from bathing beaches in southwestern Greece with 234 samples, showed that approximately 8% of them did not meet the EU criteria for the presence of *E. coli*. In the following research, an attempt was made to predict the presence of *Salmonella* sp in seawater through the presence of faecal and total coliforms. In particular, 80% of the samples (192/240) were positive for the presence of faecal coliforms from seawater samples collected in the Athens area [27,28,29,30].

In conclusion, three case studies after 2000, one Greek study published in 2018 and one from Turkey the following year, examined the microbiological quality of coastal waters in the Aegean and the Ionian seas and none of the seawater samples exceeded the limits set by the Directive 2006/7/EU. On the contrary, in the latter study, *E. coli* presence was detected above the microbiological quality criterion of the Turkish government, which is set for *E. coli* at 500 cfu/100 mL. In a thorough study of the last decade, 2149 seawater bathing areas in Greece were studied in a nine-year period (1997–2006). The authors use statistical methods, such as cluster analysis and discriminant analysis, to underline the use of only *E. coli* and enterococci for characterising the microbiological quality of coastal waters and reducing the analysis costs in public health agencies [31,32,33]. Table 1 lists case studies that applied statistical methods and models towards water quality monitoring and respective physicochemical and microbial indicators. Most of the works in Table 1 apply linear regression models to various concepts of coastal water quality. An initial initiative in Greece focused on Salmonella sp., Candida albicans and *E. coli* in coastal waters using parametric and non-parametric statistical tests. Analysis of Variance (ANOVA), the Kruskal–Wallis test, chi-square test and Spearman correlation analysis are at the frontline of statistical methods to assess microbial load. More sophisticated statistical and modelling techniques are utilised when considering spatial and temporal parameters, like artificial neural networks, principal component analysis, cluster, factor and discriminant analysis and multiple linear regression models. In parallel with pathogens’ prevalence in coastal waters, researchers spotlighted other factors, like virological quality, the potential presence of faecal streptococcus, somatic coliphages, F-specific RNA bacteriophages, faecal coliforms, faecal gene markers and antibiotic resistance genes.

In a detailed report of the 250 coastal water bodies in Greece, Greece has the longest coastline in Europe, exceeding 15,000 km; attention is drawn to the chemical and ecological profile of the water bodies in every region, yet data need to be systematically recorded for microbial quality [34].

The primary challenge lies in the collection of extensive data regarding the presence, concentration and spatial-temporal fluctuations of pathogens in coastal waters. This necessitates the mandatory monitoring of their microbiological quality. However, there is a scarcity of recent studies addressing this issue, despite the significant importance of seawater quality in Greece for tourism and cultural reasons. Hence, there is a pressing need for a systematic endeavor to comprehensively document the coastal waters in this region of Greece using advanced predictive tools such as machine learning models and classification algorithms.

None of the above research used machine learning algorithms to predict coastal water quality in Greece. Some early forms of forecasting and modelling were used, such as linear regression and principal component analysis. Although the specific works have contributed a lot to our knowledge on the quality of the Greek seas, this work is the first, to the best of our knowledge, that attempts a specific predictive approach using machine learning methods. In summary, the lack of literature on this topic, the lack of application of machine learning methods in coastal water quality and the lack of a systematic plan for the monitoring and surveillance of the EMT coastal waters, the management and exploitation of which contribute dynamically economically and culturally to the region and to Greece, piqued our research interest.

The primary objective of this study was to assess the quality status of the coastal waters of the region of Eastern Macedonian and Thrace, Greece, from 2009 to 2021, using *E. coli* load and weather parameters, with the aid of machine learning models. A further goal was to apply the k-fold cross-validation method to estimate the performance of the utilised machine learning algorithms and compare the performance with the specific data, split with 70% of the dataset dedicated to the training and the rest, 30%, to the test set. Moreover, to the best of our knowledge, there is no previous research developing predictive models to estimate coastal water quality in Greece, specifically in this region. The subsequent sections of the article are organised as follows: Section 2 provides comprehensive details about the study area, including data collection and methodology, the implementation of the machine learning models, individual performance evaluation and the validation process. Section 3 presents a detailed analysis of the data and presents the obtained results. In Section 4, these results are compared with the existing literature. Finally, Section 5 summarises the key conclusions derived from the study.

## 3. Methods

**Figure 1:**
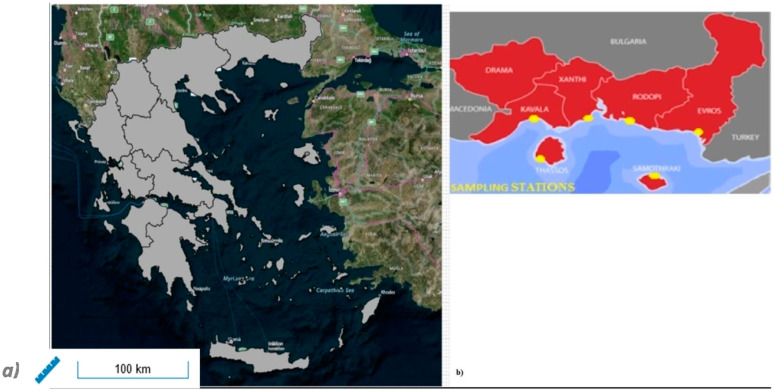
(**a**) Sampling area and (**b**) sampling stations (Source: http://geodata.gov.gr/ accessed on 1 April 2023).

Study Area and Sampling Stations: The region of Eastern Macedonia and Thrace (EMT) is situated at the northeastern edge of the country, namely the eastern part of Macedonia and the whole of Thrace. It also includes two major islands of the Thracian Sea, Thassos and Samothraki (https://www.enpe.gr/en/perifereia-anatolikis-makedonias-thrakis-en (accessed on 10 May 2023)). EMT accommodates a relatively cool climate. The weather closest to the sea is mild and Mediterranean. The temperature can drop below zero in the northern regions of EMT during winter, with lows of −5 °C recorded in recent years. In the summer period, temperature ranges typically between 30 and 35 °C. There is 100 km of rocky high-coastal area along the region’s coastline, and 70 km of hilly mid-height coast. Moreover, there is also approximately 110 km of sandy low-coast, including four river deltas (Strymonas, Nestos, Filiuri-Lissos and Evros) and lagoons. Therefore, six sampling stations in EMT, situated on the beaches of the regional units of Kavala, Xanthi, Rhodopi and Evros and the islands of Thasos and Samothraki, were selected to enter our study (Figure 1a,b). All 1039 samples were collected from May to September within a 14-year follow-up period (2009–2021). The spotted monitoring sites were used for sampling and measurements concerning the region’s coastal waters. They offer safe access to the sea, and the staff can carry the equipment and carry out the measurements. 

Meteorological parameters: Weather parameters, including average daily temperature (temperature °C), relative humidity (%) and precipitation on sampling collection day (YES/NO) and on the previous day (YES/NO), were acquired from the nearby meteorological stations (https://w1.meteo.gr/Gmap.cfm accessed on 1 April 2023). The network of automatic stations measured all basic meteorological parameters, i.e., pressure, temperature, humidity, rainfall, direction and wind strength. All the stations mentioned above were located a few kilometres from the sampling points, and they represented the weather conditions and the respective climate data from each region.

Microbiological analyses: Samples were analysed for the detection of *E. coli* according to the relevant International Organisation for Standardisation guidelines, namely the ISO 9308-1 (International Organisation for Standardisation, 2006) (https://www.iso.org/standard/55832.html (accessed on 11 May 2022)). Most samples were taken between 11:30 (a.m.) and 18:00 (p.m.), as this is when most people engaged in swimming activities. A volume of 500 mL of water was collected in sterile bottles. Samples were taken 20–30 cm below the water surface level at a sea depth of 0.8–1.3 m and were then transferred to the laboratory at 4 ± 1 °C. All samples were stored in the laboratory at 4 ± 1 °C until analyses were complete, which was always within 24 h of sample collection. Single sterile 0.45 µm pores filter disks (Pall Corporation) were placed in each filtration unit to filter 100 mL of each water sample. After filtration, the membranes were placed on CM1205B Chromogenic Coliform Agar (OXOID) plates, ensuring no air was trapped underneath dishes and incubated at (36 ± 2) °C for (21 ± 3) h. All colonies giving a positive β-D-galactosidase and β-D-glucuronidase reaction (dark-blue to violet) were considered positive for *E. coli*. Confirmatory tests were carried out as dictated by the corresponding ISO. Results were expressed as colony forming unit (cfu) per unit of volume, or log cfu per unit of volume, namely, *E. coli* (cfu/100 mL) or log *E. coli* (cfu/100 mL).

Data analysis: Statistical analysis was performed by SPSS v.21 statistical software (SPSS Inc., Chicago, IL, USA). For seasonal analysis, seasons were defined according to standard definitions in Greece (Winter: 1 December –28 February; Spring: 1 March –31 May; Summer: 1 June–31 August; and Fall: 1 September–31 November). Since the data failed to meet the assumption of normal distribution (Kolmogorov–Smirnov test) for *E. coli* and weather variables, medians and ranges were used for descriptive purposes. Spearman’s rank correlation coefficient was estimated to assess the association between the concentration of *E. coli* cfu/100 mL and meteorological parameters. Furthermore, seasonal and spatial variation of log *E. coli.* concentration were statistically evaluated by applying the Kruskal–Wallis test, a non-parametric test analogous to the analysis of variance. Unless stated otherwise, statistical tests were performed at a significance level of 0.05. 

Modelling and Experiment set up: The coastal water samples collected were classified into three distinct quality categories—‘excellent’, ‘good’ and ‘sufficient’—based on the criteria outlined in the 2006 European Directive. In the subsequent analysis, we merged the ‘good’ and ‘sufficient’ categories since they share the same *E. coli* limits, differing only in the percentile evaluation, as depicted in Table 2. The classification of coastal water quality was then simplified into a binomial characterisation, consisting of an ‘excellent’ category and another category combining ‘good’ and ‘sufficient’.

**Figure 2:**
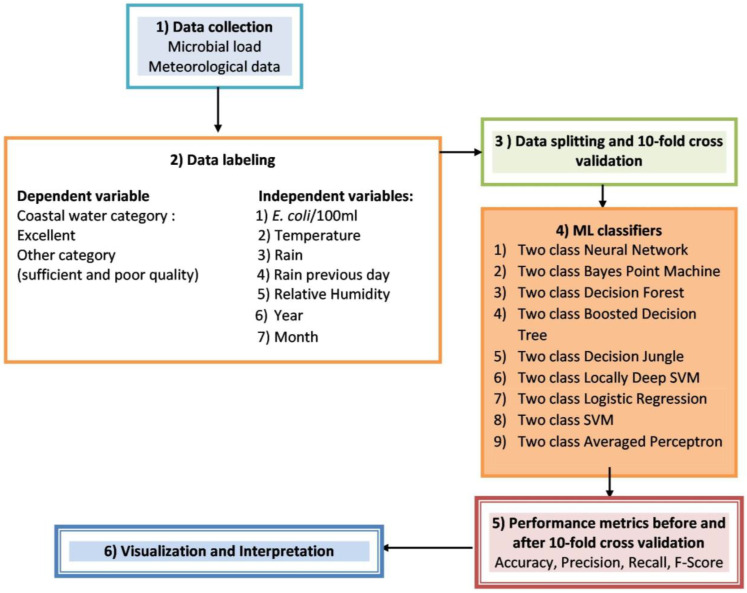
Workflow of the proposed methodology.

By employing machine learning methods, the challenge of distinguishing water quality was transformed into a data categorisation problem. In the next stage of analysis, we focused on the ‘excellent’ category and the ‘other category’, which included bathing waters of ‘sufficient’ or ‘poor’ quality. These two categories were selected to create and compare machine learning classifiers, resulting in a dataset of 825 records. Features used as inputs were: month, temperature, relative humidity, year, rain and rain the previous day. The month was categorical, temperature (T^0^) year and relative humidity (%) were continuous, while rain (yes/no) and rain the previous day (yes/no) features were nominal. *E. coli*/100 mL feature was numerical. The total number of inputs was seven. A binomial characterisation also stood for coastal water quality classification, ‘excellent’ or ‘other category’ (Figure 2). 

Automated machine learning platforms can build machine-learning models without coding. They input the dataset, problem class, evaluation metric and prediction target. They automatically execute steps of data preprocessing, feature selection and engineering, algorithm selection (clustering, classification), model training, testing and hyperparameter tuning. Subsequently, prediction models were developed in Microsoft Machine Learning Studio (Classic) to classify the above-mentioned coastal water categories, ‘excellent’ or ‘other category’. Classification algorithms are usually applied to water quality, such as logistic regression (LR), support vector machine (SVM), random forest (RF), stochastic gradient descent (SGD) and ensemble classifiers [35,36,37]. 

Logistic regression can be applied to classification tests by predicting the binary-dependent variable from a set of independent variables. Another binary, non-probabilistic classifier is a support vector machine which relies on kernel mapping. Furthermore, the random forest algorithm produces multiple trees, each constructed using a random subset of the vector features. The decisions of each tree are synthesised utilising an algorithm that gives the outcome. Two-class machine learning classifiers implemented in this study were: neural network, Bayes point machine, decision forest, boosted decision tree, averaged perceptron, logistic regression, decision jungle, support vector machine (SVM) and local deep SVM, all of which develop a binary classification model [36,37,38].

Validation: In this study, two-class machine learning algorithms, specifically classifiers, were developed to classify coastal water quality using *E. coli* and weather variables. In this analysis, 70% of the dataset was used to train the models and 30% for test purposes. In parallel, we chose to carry out cross-validation to strengthen part of the development of the mathematical models and to avoid overfitting or underfitting problems. This particular technique is followed in machine learning in order to evaluate the reliability of a model trained from a set of data and to control the variability of this data (Microsoft Azure Machine Learning documentation; Cross Validate Model).

Specifically, the K-fold cross-validation technique was adopted because it is one of the most common approaches. The model was trained using an exclusive combination of K-1 subsets of data and tested on the remaining subset. In the k-fold cross-validation, the training dataset was divided into K subsets of equal size which, in this study, equalled ten. Subsequently, ten models were generated for each subset of training data and evaluated by averaging the performance metric values of the models, i.e., accuracy, precision, recall and F1 score [6,39] (Figure 2).

The overall statistical conduct of machine learning classifiers is appraised with the aid of respective parameters, the most popular being the accuracy (1), precision (2), recall (3) and F1 score (4). These metrics are composed of TP, TN, FP and FN values representing true positive, true negative, false positive and false negative values in a produced confusion matrix [27]. To compare the modelled probability of water category classification with the described binary discrimination, a threshold of 50% probability was assumed, i.e., when modelled probability equalled or exceeded 0.50, the coastal water category was then regarded as ‘other category’. The equations of the evaluation parameters are [40,41]:

(1) Accuracy = (TP + TN)/(TP + TN + FP + FN)
(2) Precision = TP/(TP + FP)
(3) Recall = TP/(TP + FN)
(4) F1 score = 2 × (Precision × Recall)/(Precision + Recall)

## 4. Results and Discussion

### 4.1. E. coli Load and Coastal Water Quality

**Figure 3:**
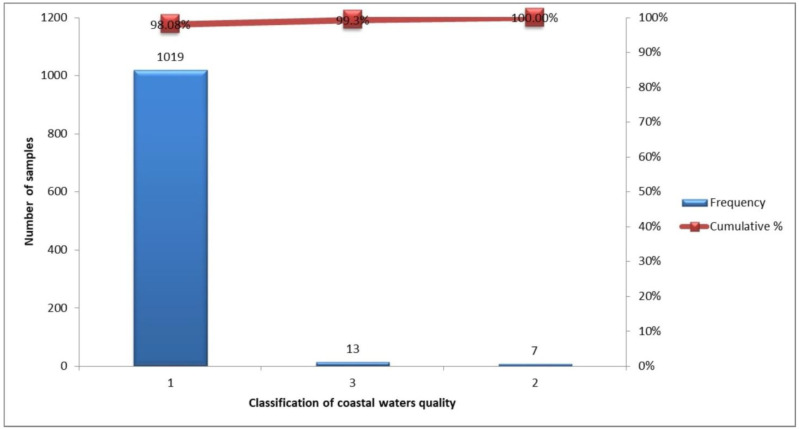
Number of samples per coastal waters category under the DIRECTIVE 2006/7/EC in East Macedonia and Thrace from 2007 to 2021 (1 = Excellent quality, 2 = Good quality, 3 = Sufficient).

The *E. coli* load in coastal waters and meteorological parameters are illustrated in Table 3 and Figure 3. Since the parameters failed to meet the normality criterion according to the Kolmogorov–Smirnov test, the median and range values are documented for descriptive purposes. From the year 2009 to the year 2013, the concentration of *E. coli* fluctuates at low levels, and the median value is zero. The years 2015 and 2021 showed the highest intermediate values of the particular pathogen. In addition, the most significant value range of *E. coli* was presented in 2009 and 2013. Finally, from 2017 onwards, high-value ranges in temperature and relative humidity were recorded in the sampling period (Table 3).

Farrel ML et al. (2021) emphasised the potential hazard arising from the emergence of ARG in bathing waters in Europe. Several studies included water quality categories for waterborne organisms of public health concern (WOPHC). Moreover, WOPHC bacteria were isolated in 35% of the examined samples, while antimicrobial-resistant microorganisms had a 47% detection rate. Five studies examining 150 samples revealed the detection of New Delhi metallo-beta-lactamase (NDM)-producing *E. coli*, *K. pneumonia* and antibiotic-resistant *E. coli*. This outcome corresponded to ‘excellent’ coastal water quality, which means a potential hazard even in the highest coastal water quality. It should be cited that, in our research, 98% of the samples were classified in this category. This finding further emphasises the rational integration into the research protocols of not only the quantitative detection of the specific microorganism but also its antibiotic resistance [42]. 

In Figure 3, the classification of coastal waters of the EMT is shown. The vast majority of the samples fall into category 1, which marks the high quality of the coastal waters of the studied region. Moreover, only 13 out of 1039 samples were classified in category 3, showing acceptable water quality standards concerning *E. coli* loads.

**Figure 4:**
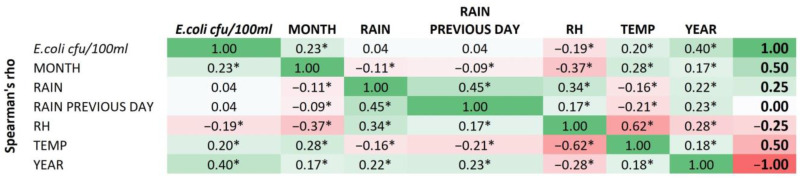
Heatmap of correlations between *E. coli* concentration and meteorological parameters. * Correlation is significant at 0.01 level (2-tailed).

Weak positive correlations were revealed between the concentration of *E.coli* with the rain parameter and the presence or absence of rain the previous day. Both of the above correlations, apart from being weak, are also notably statistically insignificant. As expected, the two specific variables are statistically significantly correlated (0.45). A negative correlation occurs between rain, month and temperature, of a mild intensity. Also, the parameter of rain on the previous day shows the same pattern as the parameter of rain: a statistically significant relationship, of a mild intensity. Finally, the variables related to time, i.e., the month and the year, show the same course, since they are significantly related to all the other parameters and, indeed, have a statistically significant relationship. Notably, month is negatively related to rain, rain the previous day and relative humidity, while the year is negatively related only to relative humidity (Figure 4).

The presence of *E. coli* and coliforms in watery ecosystems like ponds, canals, lakes and rivers may indicate the presence of other pathogens, microorganisms, protozoa and viruses [43]. The temporal variation of microbial load, *E. coli*, in a river ecosystem was also observed between February and September, mainly in the dry period. In addition, sensitivity to seasonality was also noticed for various chemical parameters like pH, phosphorus, conductivity and nitrate [44].

In a study of bathing water quality in a Mediterranean basin country, Italy, only a few samples exceeded the limit values established by the current regulation. This particular study attempted to validate the burden of *E. coli* in coastal waters and to highlight population density as the leading contributing factor of water contamination. Thus, it is suggested to conduct a thorough analysis prior to the selection of beach sampling points, considering the unique characteristics of each year and coastal area separately. 

A study of Bulgarian recreational waters also confirmed the detection of *E. coli* and other faecal coliforms by European quality standards. This study highlighted human activities’ potential hygiene pressure. These results align with our findings on *E. coli* load in coastal waters [45,46].

Precipitation has been confirmed, in other research, as a factor affecting the microbial concentration in rivers and coastal waters. Moreover, tides, water temperature and salinity also play an essential role in the presence of pathogenic microorganisms (faecal coliforms, *C. perfringens*, *Enterococcus* sp.) [47]. As stated earlier, factors such as the month, rainfall and conditions from the previous day can significantly impact the physicochemical properties of the aquatic ecosystem. These variations have the potential to pose risks and adversely affect the overall water quality. Researchers in another study acknowledged the precipitation factor when predicting recreational water quality. Specifically, the precipitation level of the last two days was one of the factors influencing enterococci presence in recreational waters in Puerto Rico [48].

**Figure 5:**
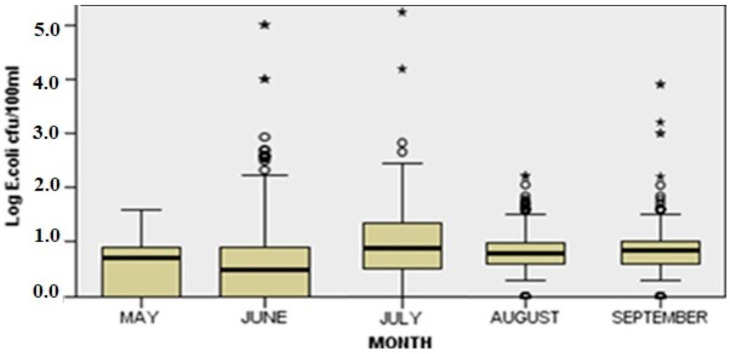
Seasonal variation of *E. coli* in all sampling sites (The box lines indicate the lower and upper quartiles and the central line is the median. The whiskers show the minimum and maximum values. Circles represent outliers and asterisks extreme outliers).

**Figure 6:**
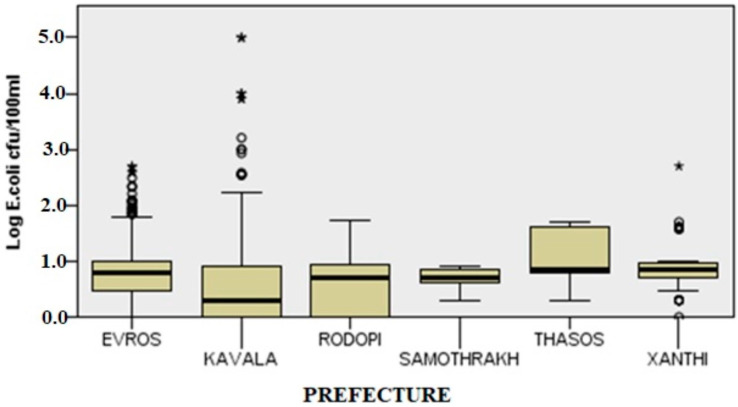
Spatial variation of *E. coli* in all sampling sites (The box lines indicate the lower and upper quartiles and the central line is the median. The whiskers show the minimum and maximum values. Circles represent outliers and asterisks extreme outliers).

In Figure 5 and Figure 6, the seasonal and spatial variation of log *E. coli* concentration between sampling points and months are illustrated. Seasonal fluctuations are present since the distribution of *E. coli* is not the same across months. The applied Kruskal–Wallis test showed that there were statistically significant differences in *E. coli* load among months (H(4) = 52.679, *p* = 0.000). 

The applied Kruskal–Wallis test showed that there were statistically significant differences in *E. coli* load among sampling locations (H(5) = 68.431, *p* = 0.000). 

Using pairwise comparisons, it was also possible to illustrate spatial variations by site in the prefecture of East Macedonia and Thrace. Specifically, Kavala-Evros, Kavala-Xanthi and Kavala-Thasos, which belong to neighbouring geographical divisions, demonstrated significant differences in *E. coli* load (*p*-value < 0.05). Our study found that the remaining combinations of regions did not exhibit statistically significant differences. While some of these combinations involved neighbouring regions, the analysis did not reveal any significant associations. Furthermore, when examining the two islands in the East Macedonia and Thrace region, Samothrakh and Thasos, we observed that they were not strongly associated with the other regions in the area, except for a spatial association between Kavala and Thasos. Additionally, it is worth noting that the regions located at the edges of the East Macedonia and Thrace regions, namely Kavala bordering Central Macedonia and Evros bordering Turkey, exhibited statistically significant relationships. These findings indicate distinct patterns of regional relationships within the study area, with some regions showing closer associations due to geographic proximity or shared borders with other regions. 

Indeed, specific local conditions, including different land uses and various water ecosystems such as rivers, lakes and lagoons, can contribute to the observed variations in pathogen concentration and their detection in coastal waters. These factors can influence the transport and dissemination of pathogens and their survival and persistence in different environments. For example, areas with intensive agricultural activities or urban development may have increased inputs of pollutants and contaminants into water bodies, affecting water quality and potentially contributing to higher pathogen loads. Similarly, regions with significant river systems or interconnected waterways may experience increased pathogen inputs from upstream sources, leading to variations in pathogen concentrations along the coast. Factors such as tidal patterns, water circulation, and coastal morphology can also play a role in the distribution and dispersion of pathogens in coastal waters. These factors can create localised conditions that favour the proliferation or decay of pathogens, leading to spatial variations in their concentrations [49,50,51,52,53,54,55].

The components of temperature and precipitation have considerably affected the quality of marine waters in different latitudes and longitudes. Seasonal variation of temperature, shifts in rain flow, precipitation impact on the salinity level and the diffusion of various streams and rivers into coastal waters also affect their quality status at a chemical and microbiological level, both seasonally and annually [41,56]. 

*E. coli*’s seasonal prevalence in coastal waters has been confirmed in Greece and in our research. Particularly, the pathogen distribution is divided into three distinct patterns; the first pattern appears in May, the second from June to August and the third in September and October [28,33]. According to our statistical analysis, the month and the year contribute to various the *E. coli* loads in coastal waters. 

Climate change exerts both direct and indirect effects on the concentration of pathogens, such as *E. coli.* It influences various climate-related phenomena, including rising seawater temperatures, fluctuations in precipitation, intensified wave activity and increased wind speeds. These alterations disrupt the physicochemical parameters of aquatic ecosystems, particularly in coastal waters and sand, leading to variations in the prevalence of microorganisms [57].

Furthermore, climate change contributes to an elevated presence of thermo-tolerant microorganisms, an increased microbial load in sand and beaches caused by the influx of visitors and the adaptation of specific pathogens to drought conditions. These impacts have significant implications for water body hygiene and pose health hazards. As air and sea temperatures continue to rise, extreme precipitation events become more frequent, sea levels increase and there are changes in sea salinity and water activity; the risks associated with waterborne pathogens escalate [57]. 

In our study, we mainly focused on several weather factors. Temperature and relative humidity influenced pathogen distribution and both are statistically significant. The conjunction and interrelation of water quality and climate variability are well established, for example, rainfall and temperature fluctuation in spatial and temporal scales. Nijhawan A. and Howard G. (2022) highlighted the role and association of climate variables, the prevalence of microbial concentrations and, specifically, microbial pathogens in low- and middle-income countries (LMICs). Factors like precipitation index and heavy rainfall (>10 mm), in the period of the two weeks before sampling, are good predictors of *E. coli* and water quality. Another component regarding the period before sampling, and its positive influence on pathogen concentration, is the number of hot days (days with temperature in the 90th percentile of reference data) [58].

### 4.2. Predictive Model Performance

This study evaluated nine classification techniques and the respective evaluation metrics, namely two-class classifiers: neural network, averaged perceptron, logistic regression, support vector machine, locally deep support vector machine, Bayes point machine, decision forest, boosted decision tree and decision jungle.

In Table 4, a comparison of the proposed classifiers is outlined. Two-class decision forest, decision jungle, locally deep SVM and boosted decision tree performed better than the other classifiers. Notably, these four had almost the same score in accuracy (100%), area under curve (AUC), F1 score (100%), precision (100%) and recall (100%). In addition, all other models performed with 98–99% accuracy. In the metric value of precision, the models achieved values of 100%. In the recall assessment, Bayes point machine had the poorest performance (0%), logistic regression, SVM, locally deep SVM and averaged perceptron achieved 60%. The rest of the classifiers reached 100% and neural network achieved 80%. Finally, for the F1 score, Bayes point machine had 0%, while SVM, locally deep SVM, logistic regression and averaged perceptron accomplished 75%. The remaining values in this category for the other classifying models were 100%. The AUC reached 100% for all models except the Bayes point machine.

The following table illustrates the cross-validation performance of the selected models considering the average score of ten folds for every metric value: accuracy, precision, recall, AUC and F1 score (Table 5). Regarding accuracy, all models achieved a high score; three out of nine scored 100%. However, in terms of the other metrics, some models were superior to others. In particular, Bayes point machine scored the lowest, with only 20% precision. In the recall metric value, the same classifier scored a single-digit percentage, while logistic regression achieved the second lowest value, 48.33%. Averaged perceptron reached almost 57%. Bayes point machine and logistic regression are grouped in the last two positions in the list concerning the F1 score. In this vein, it is depicted that 10-fold cross-validation altered the score sorting of some models.

Remarkably, after cross-validation, three out of nine models achieved perfect accuracy performance, namely 100%, compared to four out of nine models of the classic split method of the dataset, 70% training and 30% testing. In terms of precision score, the classical method outperforms the 10-fold cross-validation approach. The 10-fold cross-validation, in contrast, had mixed results ranging from 20% to 90%. The recall values in the classical method included three classifiers with 100% scores and none during cross-validation. In addition, the last method encompasses a classifier with a 7% score, Bayes point machine. The performance of the models, as indicated by the area under the curve metric, experienced a slight decrease in comparison to the validated results. Bayes point machine consistently displayed the lowest value, while the other classifiers achieved satisfactory results of approximately 90%. This observed decline in model performance after conducting 10-fold cross-validation is consistent with the findings of a separate study that investigated the prediction of salt concentration in water samples. The study identified a decision forest algorithm as the most effective classifier in that context [59].

On the contrary, the classical method had an F1 score of 0% in a machine learning classifier, the two-class Bayes point machine. Finally, in terms of the F1 value, there was a broad spectrum of scores in the performance of the models. For example, Bayes point machine scored 0% in the classical method and 10% in 10-k validation. They were followed by decision forest, boosted decision tree and decision jungle, with 100% in the classical and 90% in cross-validation. Overall, the two-class machine learning classifiers, decision forest, decision jungle and boosted decision tree, were superior in most metrics to all others in both methods, specifically after considering the F1 value after the 10-fold cross-validation of the models. F1 scores provide valuable insights as a performance metric in binary classification [60]. 

The versatility and superiority of decision forest algorithms were outlined in a recent study estimating arsenic contamination in groundwater samples. A differentiated form of the decision forest algorithm was used to predict arsenic in groundwater with high accuracy and recall values [61]. In addition, *E. coli* prediction in a watershed, with the aid of the random forest algorithm, was the outcome of dela Pena et al. (2021). This result was the combination of applying machine learning algorithms and molecular tools to estimate faecal coliform load, which aligns with the results of our performance metrics of the random forest algorithm after cross-validation [62]. 

After considering the performance results of the models, their interpretation should not be strictly made with only one metric, e.g., accuracy. The critical prediction issue, i.e., the actual risk stemming from a high false negative event (FN) rate versus a high false positive (FP) rate, should also be considered. The actual health risk is assessed with each case’s existing conditions and requirements. Health authorities should prioritise the false negative rate when it comes to swimming [63,64]. 

Machine learning, as a subset of artificial intelligence, is a new concept in several aspects of water quality and resources management. The essential topics discussed and analysed in the current literature are modelling, prediction and forecasting, decision support, operational management and optimisation [65].

Articles involving mathematical models and machine learning algorithms in water science were evaluated in a review paper in ‘Water Resources’. This research revealed that about 5500 articles used ANNs, followed by 1127 that utilised support vector machines or support vector regression, 1360 that used decision trees, regression trees or random forests, and 85 Bayesian networks. Additionally, only 1% of these applications focused on water quality modelling issues instead of other categories, such as water treatment and distribution [35]. 

Moreover, random forest, support vector machines and classification/regression trees, as implemented in our analysis, were also the leading algorithms in complex environmental studies. These applications examined the infiltration rates of permeable stormwater channels, faecal indicator prediction in bathing waters, faecal contamination in environmental samples, conventional water quality indices, water quality in constructed wetlands, water quality index, level of algal bloom in reservoirs and seasonal variability of *E. coli* in irrigation ponds [66].

Today’s modern context of water quality management requires the full exploitation of the capabilities offered by the development of computational power and the introduction of machine learning and regression models (random forest, artificial neural network). The real-time exploitation of field data and incorporation of environmental and meteorological factors in models produce large data sets. Thus, predicting water quality categorisation from historical data can now be feasible, primarily when classification is based on international indicators, regulations and on holistic approaches to assessing risks to and impact on human health [67].

Least-angle regression site-specific predictive models were developed to forecast *E. coli* load on three beaches in the USA. The researchers reported that predictive performance (cross-validation) was significant only for qPCR-based enterococci. At the same time, weather variables like antecedent rainfall, wave height and wind speed/direction were dominant across all models [40]. In our research effort, the factors of rain, temperature, relative humidity and rain in the previous day did not play a vital role in *E. coli* abundance in the coastal waters of EMT. 

Additional research efforts towards the real-time modelling of *E. coli* regarding water quality suggest the usage of Bayesian belief networks (BBNs). This modelling proposal aimed to overcome the intricate relationships between environmental, meteorological and microbiological factors. BBNs elevated prediction accuracy by 25–54% compared to other techniques like random forests, logistic regression and naïve Bayes. Moreover, forecasting *E. coli* load within a binary classification plan was notably enhanced using BBNs with cross-validation, completing prediction accuracies over 80% for all locations [68]. Our results also showed that Bayes point machine and logistic regression algorithms scored the lowest accuracy and F1 values. 

A machine learning model, an advanced random forest regression version, was utilised to describe the link between water quality and environmental factors. A 10-fold validation technique, as in our study, was deployed, and the research outcome was better than the multiple linear regression and geographically weighted regression models previously exploited in the Chesapeake Bay watershed [69]. In line with our results, 10-fold cross-validation enhanced the predicting capability of the models. 

The random forest algorithm, employing 10-fold validation, outperformed six other algorithms in predicting water quality based on microbial distribution. These algorithms included ANN, multinomial logit, naïve Bayes, k-nearest neighbor, support vector machine and linear discriminant analysis. Our study found that decision forest and boosted decision tree yielded better results compared to the other algorithms. Another study conducted within the same framework, focusing on water quality, utilised the random forest regression model with 5-fold validation, demonstrating a higher accuracy score when predicting the permanganate index and total phosphorus in an aquatic ecosystem [70,71]. 

One of the main problems in the mathematical prediction of the quality of aquatic ecosystems, including recreational waters and swimming beaches, is the high variability that occurs at spatial and temporal scales. Wang L. et al. (2021) applied a technique in which machine learning algorithms (partial least square, sparse partial least square, multiple linear regression, random forest, Bayesian network) were used as input data in a final prediction model. A leave-one-year-out cross-validation method was implemented. This particular method achieved high rates of accuracy in modelling the evaluation of beach water quality of three swimming beaches in the United States: 78%, 81%, and 82.3%, respectively [72]. A high modelling performance was also observed when employing a 10-fold cross-validation technique (neural network and decision jungle). The same outcome was noticed in our probe when three out of nine models achieved perfect accuracy after cross-validation. Furthermore, predictive linear regression models, that support the creation of probability maps, are likewise pertinent. Predictive models can be used for ‘what-if’ cases in order to manage watery ecosystems and respond to various ecological burdens [73]. 

An alternative prediction method for water quality, taking into account the water quality index, deep learning and auto deep learning techniques, is presented by Prasad et al. (2022). After splitting the dataset into a 4:1 ratio for training and testing procedures, various algorithms are compared for binary and multiclass water quality classification. Briefly, conventional deep learning performed better than auto deep learning for binary and multiclass classification. Artificial neural networks achieved 86% and 77%, recurrent neural networks generated 87% and 89%, and long short-term memory scored 92% and 94% for binary and multiclass classification, respectively [74]. As can be seen manifested in our results, decision forest, decision jungle and boosted decision tree achieved satisfactory scores in accuracy and precision metrics with and without cross-validation. Furthermore, artificial neural network and support vector machine algorithms demonstrated inflated accuracy scores in Malaysia’s river classification problem for wet and dry seasons [75]. 

Table 6 provides a basic overview of compared metrics, considering the machine learning classifiers discussed in this section. The best classifier is presented, as well as the respective metric determined in the study, and is compared with the evaluation metric of accuracy in this current study.

The presence of pathogenic microorganisms of faecal origin in coastal and swimming waters not only poses risks to swimmers and visitors but also has implications for the spread of antibiotic-resistant genes in these areas. In addition, the antibiotics used in fish farming pose an additional risk to the entire aquatic ecosystem and its organisms. On a global scale, 45 antibiotic substances used in fish farming systems have been identified and their persistent use, along with environmental and chemical factors such as sunlight, salinity, minerals and DOM, can exacerbate and degrade the ecological status of marine microorganisms (toxicity, bioaccumulation and proliferation of resistant genes). We attempted to predict their distribution on the coast after rainfall with the help of machine learning models, namely conventional long short-term memory (LSTM), a LSTM–convolutional neural network (CNN) hybrid model and input attention (IA)-LSTM. According to this research, the IA-LSTM performed very well [76,77]. In this contribution, the neural network model scored good performance metrics like accuracy and F1 value. 

The link between antibiotic-resistant genes, environmental contamination and the impact on marine and aquatic ecosystems must be explored and constantly evaluated, as stated by the experts of the World Health Organisation and the Health-Related Water Microbiology Specialist Group (HRWM-SG) of the International Water Association (IWA) [78,79].

### 4.3. Study Limitations

It is important to acknowledge certain limitations when drawing conclusions about the performance of our models and comparing evaluation metrics with other studies conducted. A limitation of this work was the absence of extra weather parameters, such as solar radiation, or the testing of these parameters and the *E. coli* load in other water bodies. In addition, an area that warrants attention in future research endeavors is the estimation of uncertainty in water quality prediction models, which will be our primary focus.

In conclusion, the monitoring of coastal waters exclusively during the swimming season provides only a restricted view of water quality. To obtain a more comprehensive understanding, it is advisable to implement a comprehensive and consistent annual monitoring program. This approach would enable a better assessment of the temporal microbial load and account for variations between different sites. When conducting longitudinal studies, it would be valuable to consider the factors associated with urban development in coastal zones, such as population size, land use and wastewater treatment plants. The overall surveillance of EMT coastal waters on an annual basis, and on more sampling areas, would give a detailed picture of the microbiological quality of coastal waters and improve the models’ applicability, deployment and performance.

## 5. Conclusions

In order to reliably determine a public health outcome, it is advisable to apply a combination of methods and use data from various sources, e.g., meteorological data, microbiological methods and mathematical models. Such synergistic approaches are applied to categorise water quality for various watery ecosystems, such as drinking water, rivers and coastal waters.

A basic understanding of the current status of modelling across different weather parameters and microbial load, namely *E. coli* concentration in NE Greece and the issues associated with contemporary machine learning algorithms, are presented in this research paper.

This study implemented machine learning algorithms to predict the category classification of coastal waters in EMT, with *E. coli* and weather variables. Based on the satisfactory results before and after cross-validation, machine learning approaches, like two-class decision forest, decision jungle and boosted decision tree, can provide critical supplementary information about the dynamic of *E. coli* contamination and, concurrently, consider the meteorological parameters for coastal water classification. 

This research provides an additional contribution by proposing a new approach to validation in machine learning algorithms. It offers a validation perspective that can guide future research in developing predictive models for categorising coastal waters based on the existing regulations for pathogenic microbial load.

To advance our understanding, the categorisation of coastal water quality using modeling techniques necessitates collaborative studies that involve experts from diverse fields including epidemiology, hygiene, mathematics, data science and molecular biology, among others. By fostering interdisciplinary collaboration, we can achieve further progress in this area. Moreover, it is valuable to sustain research initiatives focused on monitoring coastal water in the future, particularly in countries where the tourism industry plays a crucial role and where coastal regions experience high levels of overcrowding during the summer season. Such endeavors will lead to additional insights and improvements in managing coastal water resources.

This approach aims to enhance the decision-making processes of regional and national agencies concerning coastal, environmental and hygiene management. Overall, further research efforts are required to advance the algorithms and predicting models for predicting pathogens and assessing the quality of coastal waters. Machine learning models can be a vital supplementary tool for water quality management planning.

## Figures and Tables

**Table 1:** Statistical methods and applications.

Model	Work Applied	References
Multiple linear regression model–artificial neural network (ANN)	Daily prediction of beach water quality—*E. coli* concentration	[20]
Linear mixed model	Linear mixed model presenting the relationship between life satisfaction and objective water quality	[22]
ANOVA–chi square correlation analysis	Assessing *Salmonella*, *Campylobaeter* and *Yersinia* spp. occurence in river and lake samples’	[23]
Mann–Whitney U–Wicoxon Rank Sum W test	presence of *Listeria*, *Salmonella*, Total coliforms, faecal coliforms and faecal streptococci in river and lakes samples	[24]
Kruskal–Wallis test	Presence of *Salmonella* or *Candida albicans* in coastal waters	[30]
Hydrodynamic models	Water quality monitoring in the Thermaikos bay area-total coliforms, *E. coli*	[25]
Shapiro–Wilk and Spearman test	Virological and microbiological quality of marine and running surface waters	[31]
Multiple linear regression	Seasonal fluctuation of faecal coliforms, *E*. *coli*, faecal streptococci in coastal waters	[28]
ANOVA	Somatic coliphages, F-specific RNA bacteriophages, bacteriophages infecting *Bacteroides fragilis*, *Escherichia coli* and enterococci in bathing waters	[27]
Kruskal–Wallis, Chi square test, correlation analysis	Microbiological water quality of bathing sites (total coliforms, faecal coliforms, faecal streptococci, *E. coli*, somatic coliphages, F-RNA bacteriophages, bacteriophages infecting *Bacteroides fragilis*, enteroviruses, adenoviruses and hepatitis A viruses)	[29]
Cluster, factor and discriminant analysis,	Assessment and modeling of microbiological quality data concerning coastal bathing water	[33]
ANOVA, principal component analysis	Seasonal and spatial variation of faecal indicator bacteria in coastal zones	[32]
Regression–principal component analysis	Faecal marker genes and antibiotic resistance genes in stream water samples	[21]

**Table 2:** Quality categories for coastal waters and transitional waters.

Parameter	Excellent Quality	Good Quality	Sufficient	Reference Methods of Analysis
*E. coli*				ISO 9308-3 or
(cfu/100 mL)	250 ^1^	500 ^1^	500 ^2^	ISO 9308-1

^1^ Based upon a 95-percentile evaluation. ^2^ Based upon a 90-percentile evaluation.

**Table 3:** Descriptive statistics of *E. coli*, Temperature (T^0^) and Relative Humidity (RH) in coastal waters of East Macedonia and Thrace from 2009 to 2021.

Year	Median			Range			Minimum			Maximum		
	***E. coli***	**T^0^C**	**RH%**	***E. coli***	**T^0^C**	**RH%**	***E. coli***	**T^0^C**	**RH%**	***E. coli***	**T^0^C**	**RH%**
2009	0	25.9	65	100,000	10.1	28	0	19.1	40	100,000	29.2	68
2010	0	20.9	59	1000	10	30	0	15.7	48	1000	25.7	78
2011	0	19.6	78	350	6.8	22	0	19.6	56	350	26.4	78
2012	0	24.2	64	850	11.4	41	0	18	44	850	29.4	85
2013	0	25.8	43	100,000	8.9	25	0	20	43	100,000	28.9	68
2014	7	26.3	57	360	20	32	0	19.2	41	360	30.2	73
2015	15	26.4	54	100	10.2	32	0	19.2	40	100	29.4	72
2016	7	25.6	50.5	70	12.8	31	0	19.1	41	70	27.9	72
2017	7	25.7	57	500	12.8	47.3	0	17.2	39	500	30.7	78
2018	7	26.2	53.5	60	16.4	46	0	12.1	38	60	28.5	84
2019	5	23.7	70	40	17.3	50	1	12.1	35	40	29.4	85
2020	5	25.3	48	70	13.9	41	1	16.3	37	70	30.2	79
2021	30	27.8	53	500	15.9	36	3	16.3	40	500	32.2	76

**Table 4:** Metric values without cross validation.

Without Cross-Validation %					
MODEL	Accuracy	Precision	Recall	F1 Score	AUC
Neural Network	99	100	80	89	100
Bayes Point Machine	98	100	0	0	74.5
Decision Forest	100	100	100	100	100
Boosted Decision Tree	100	100	100	100	100
Decision Jungle	100	100	100	100	100
Locally Deep SVM	100	100	100	100	100
Logistic Regression	99.2	100	60	75	100
SVM	99.2	100	60	75	100
Averaged Perceptron	99.2	100	60	75	100

**Table 5:** Mean metric values after cross-validation of the models.

Final Cross-Validation 10-Fold %					
MODEL	Accuracy	Precision	Recall	F1 Score	AUC
Neural Network	99.88	90.00	86.67	88.00	90
Bayes Point Machine	97.94	20.00	7.00	10.00	61
Decision Forest	100.00	90.00	90.00	90.00	90
Boosted Decision Tree	100.00	90.00	90.00	90.00	90
Decision Jungle	100.00	90.00	90.00	90.00	90
Locally Deep SVM	99.51	80.00	76.00	78.00	90
Logistic Regression	99.15	70.00	48.33	56.00	90
SVM	99.76	80.00	76.67	78.00	90
Averaged Perceptron	99.39	70.00	56.67	61.33	90

**Table 6:** Comparison of performance metrics of different algorithms.

Classifier	Performance Metrics		References
	Comparative Study	Current Study	
Random Forest	Accuracy 90–100%	Accuracy 90–100%	[52]
Random Forest	Accuracy 75.8%	Accuracy 90–100%	[54]
Random Forest	Accuracy 7–74%	Accuracy 90–100%	[55]
Decision Tree Optimiser	4–49% correct predictions	Accuracy Boosted Decision Tree 100%	[56]
Multivariate Linear Regression	False Negative correct predictions 52–62%,	Accuracy Logistic Regression 99.15%	[57]
Random Forest	Accuracy 65%	Accuracy 90–100%	[59]
Regression models	Overall Performance = 1.87	Accuracy Logistic Regression 99.15%	[40]
Bayesian Belief Networks	Accuracy 81–95%	Accuracy 97.94%	[61]
Random Forest Regression	R^2^ coefficient of determination 79–99%	Accuracy 90–100%	[62]
Random Forest	Kappa coefficient 58–93%	Accuracy 90–100%	[63]
Random Forest Regression	R^2^ coefficient of determination 80%	Accuracy 90–100%	[65]
Model Stacking of Four Models	Accuracy 78–82.3%	Accuracy 99.15–100%	[64]
Linear Regression	Accuracy 55–73%	Accuracy 99.15%	[66]
Deep Learning Convolutional Neural Network	Accuracy 98%	Accuracy Neural Network 99.8%	[67]
Artificial Neural Network	Accuracy 88.7–89.5%	Accuracy Neural Network 99.8%	[68]

## Data Availability

https://meteo.gr/.
